# Investigating ‘Fear of Missing Out’ (FOMO) as an extrinsic motive affecting sport event consumer’s behavioral intention and FOMO-driven consumption’s influence on intrinsic rewards, extrinsic rewards, and consumer satisfaction

**DOI:** 10.1371/journal.pone.0243744

**Published:** 2020-12-14

**Authors:** Jeeyoon Kim, Younghan Lee, Mi-Lyang Kim

**Affiliations:** 1 Department of Sport Management, Falk College of Sport and Human Dynamics, Syracuse University, New York, New York, United States of America; 2 Department of Kinesiology, Mississippi State University, Starkville, Mississippi, United States of America; 3 Department of Sport, Leisure, and Recreation, Soonchunhyang University, Asan-si, Choong-nam, Korea; Aalborg University, DENMARK

## Abstract

This study posits that Fear of Missing Out (FOMO) can function as an extrinsic motive stimulating sport event consumption by inducing consumers to overcome leisure constraints. Also, FOMO-driven consumption is proposed to affect consumption experience for being grounded on extrinsic than intrinsic rewards. In Study 1, the moderation of FOMO between intrapersonal and structural constraints and sport media viewing intention are tested. In Study 2, the relations among FOMO-driven consumption, intrinsic rewards (i.e., enjoyment), extrinsic rewards (i.e., social adherence), and consumer satisfaction are assessed. Study 1 results support the notion that FOMO can boost sport media viewing intention through two mechanisms: by directly stimulating intention and by lifting the negative effect of constraints on intention. In Study 2, FOMO-driven consumption shows a stronger link to extrinsic than intrinsic rewards, extrinsic reward is marginally but negatively associated with intrinsic reward, and intrinsic reward is a stronger predictor of satisfaction. Overall, FOMO is identified as a meaningful extrinsic motive for sport event consumption though its effects on consumer satisfaction are arguable. Implications for FOMO-driven marketing are discussed.

## Introduction

Fear of missing out (“FOMO”) is “a pervasive apprehension that others might be having rewarding experiences from which one is absent” (p. 1841) [1]. As social beings, people tend to have social concerns about being uninformed and feeling left out of what many others do, and can act in ways to mitigate such concerns [2]. The phenomenon of FOMO can be found in social reports indicating that 56% of adults who use social media and 69% of millennials in the US experience FOMO [3, 4]. In Canada, 60% of millennials reported having an experience of making a reactive purchase after feeling FOMO, mostly within 24 hours, and in many cases related to events [5]. These reports indicate that FOMO is not only a popular social-cultural phenomenon but also a significant extrinsic motive for consumption behavior [6].

In the sport setting, conceptual evidences of FOMO impacting consumption behavior is spotted, particularly in the event context. For example, FOMO-related perceptions such as ‘to follow a trend’, ‘everyone else seems to do so’, and ‘it is the big game not to miss out’ are found to prompt people to consume popular sport events (e.g., the Super Bowl, the Olympics) [7, 8]. Other studies suggest that FOMO is a meaningful motive, as conforming with social others can stimulate the behavior of sport fans [9, 10]. The notion that FOMO is a form of extrinsic motive for sport event consumption can be linked to socialization studies as social apprehension and compliance are known to cause people to support a sport team popular among others [11, 12].

More specifically, based on self-determination theory (“SDT”) [13], FOMO can be understood as a social concern about a comparative deficit in competence and relatedness, which deficit comes from being non-conversant and left out from a popularly shared event [14, 15]; such concerns can socially compel and extrinsically coerce one to engage in the event [16]. Hence, FOMO as a form of extrinsic coercion can be a unique motive for sport event consumption. First, the extrinsic coercion of FOMO can be effective in enforcing one to face and overcome their hesitancy about or barriers to behavior [17]. In other words, FOMO may stimulate sport event consumption by facilitating the lifting of constraints (i.e., factors impeding one’s action; e.g., lack of interest or time) [18]. Testing the moderation of FOMO in the link from constraints to behavioral intention can advance our knowledge of FOMO and the mechanism of extrinsic motive stimulating intention. Second, the extrinsic coercion of FOMO drives one to pursue extrinsic (or instrumental) rewards such as risk avoidance and social status in the consumption experience [2, 6, 19] rather than, and possibly with the hindrance of, the intrinsic reward of pure enjoyment (based on the cognitive evaluation theory within SDT) [13]. The pursuit of extrinsic rewards shapes sport consumer’s objectives, expectations, reward attainment, and thus satisfaction in ways distinctive from the pursuit of intrinsic rewards. Investigating how the extrinsically motivated FOMO-driven behavior is associated with the sport consumer’s experience will add another avenue in understanding the full spectrum of sport event consumption.

However, empirical testing of FOMO related to sport event consumption is limited. Most studies in the sport context offer a conceptual understanding of how FOMO may affect behavioral decisions [10, 11, 12] or examples of motives related to FOMO on a social level [7, 20]. Perhaps, the work by Larkin and Fink is one of the rare studies that tested FOMO as a predictor of sport consumption but limited to fantasy sport and use of social media [21]. No studies up to date have specifically investigated FOMO as a moderator of constraints that affects sport event consumption and the pursuit of rewards in the process. Therefore, grounded in SDT, this study investigates the phenomenon of FOMO as a factor affecting behavior and experience surrounding sport event consumption. We test FOMO as an extrinsic motivation that moderates (or weakens) the negative association between constraints and behavioral intention. We further examine how FOMO-driven sport event consumption is linked to intrinsic rewards (i.e., enjoyment) and extrinsic rewards (i.e., social adherence) and thus to consumer satisfaction. The findings offer novel insights into the functionality of FOMO-driven marketing in promoting sport event consumption and enhancing sport consumer satisfaction.

### FOMO and SDT

According to SDT, people are motivated to behave in ways that satisfy three fundamental psychological needs: competence, relatedness, and autonomy. Psychological experience in an action is defined by the (un)satisfaction of these three needs [13, 22]. More specifically, individuals can be intrinsically motivated (in search of the intrinsic rewards of pure enjoyment and satisfaction) or extrinsically motivated (in pursuit of the extrinsic rewards of personally important instrumental outcomes such as reputation, status, or avoiding risks) to act in ways that satisfy the needs [13]. The type and strength of motives shape one’s goals for rewards and need satisfaction in a behavior, influencing the success/failure of goal achievement and the psychological experience during the behavior. Need satisfaction arising from intrinsic rewards are known to derive a more positive experience than that based on extrinsic rewards [22].

FOMO is a type of anxiety whereby a person becomes compulsively concerned about missed opportunities for social interactions and/or satisfying experiences [1]. SDT is a theoretical basis that is popularly used to explain FOMO as a form of apprehension about one’s deficit state of need satisfaction [1, 15]. Missing out on a popular or positive experience may lead to senses of ‘incompetence’ for hindering one’s knowledge, status, and adequacy in a society [14], and ‘lack of relatedness’ for raising concerns such as social exclusion, ostracism, and social anxiety [14, 23]. Fear of deficits related to competence and relatedness can socially compel and thereby extrinsically enforce one to be part of the experience, impeding autonomy [16].

That is, FOMO can function as an extrinsic motive that triggers behavior in avoidance of comparatively unsatisfied needs. The goal of FOMO-driven behavior is to seek extrinsic rewards that can be instrumental in the risk avoidance of incompetence and lack of relatedness [6, 15]. Focusing on and achieving extrinsic rewards can be influential on one’s psychological experience in FOMO-driven behavior. Therefore, in this study, FOMO is approached as an extrinsically imposed psychological condition or change affecting one’s decision-making for and experience during a particular sport event.

### FOMO as an extrinsic motivation

FOMO has been researched in contexts such as social media and internet usage [2, 14, 16, 23] as well as purchasing behavior [6, 17]. In business studies, FOMO-driven marketing has been investigated as a stimulant for consumption in pursuit of acquiring status, self-branding, instant gratification, social rewards, and conformity [6, 19]. FOMO has been approached as a state as well as a trait [24] for being driven by external stimulation and/or internalized self-regulation to conform with social others (based on the Organismic Integration Theory in SDT) [13]. Hodkinson noted that FOMO is distinctive from other consumer motives (e.g., joy, esteem) in that, by being grounded in fear, FOMO coerces consumers to address directly their hesitancy to take action [17]; the coercion may accompany anxiety and stress but is known to function as a strong external motive for behavior [13, 17].

Sport event consumption is a context where FOMO can affect one’s behavioral decisions. As prevalent leisure and daily conversation topics that are effective for social facilitation and in-group formation [25, 26], missing out on and being non-conversant in a popular sport event can raise concerns about being left behind with regards to relatedness and competence, resulting in FOMO. While there is a lack of research on FOMO in sport event consumption contexts, hints of FOMO are found in studies on motivation and socialization.

Knowledge acquisition, social affiliation, and community support are examples of motives relevant to FOMO, as formed based on urges to stay competent and relatable in a social group [20, 27]. In socialization studies based on self-categorization, social apprehension motivates people to follow a team supported by meaningful others (e.g., family, community) [11]; the strength of motive relies on the relative size and homogeneity of the social group [12]. In the Psychological Continuum Model [10], sport consumers in the attraction stage follow a team to conform with social others, conditional on external influences. Fan attachment can draw a sense of obligation (as a fan) based on social norms and can stimulate behavior [9]. These studies indicate social apprehension and desire to conform as factors driving sport consumption, implying FOMO as a meaningful extrinsic motive warranting attention.

Larkin and Fink’s work is perhaps the only study testing FOMO in relation to sport consumption [21]. Referring to identity theory [28], the researchers explained that an individual holds multiple identities, specific identities become activated based on given situations, and FOMO can lead to a higher probability of summoning a particular identity. With a focus on the fantasy sport context, FOMO was found to be positively associated with one’s identity salience as a sport team’s fan directly and indirectly via fantasy sport and social media involvement. In the study, FOMO was viewed as a stabilized psychological condition, and fantasy sport was discussed as a channel to attain team-related information to relieve FOMO. Such approaches avail insights into FOMO as a factor motivating behavior in sport consumption, but can be expanded to understanding the situational stimulation of FOMO [17, 24] and to the context of sport media consumption (as Larkin and Fink suggested) [21]; further research on FOMO is required.

### FOMO as a moderator lifting constraints

As an extrinsic motive, FOMO is anticipated to directly impose positive effects on sport consumption behavior. However, the effect of extrinsic motivation can be more complex. According to ‘negotiation of motives and constraints’ [29], one’s decision for leisure behavior is made based on an interaction (or negotiation) between motives and constraints. For leisure behavior, one should overcome intrapersonal (related to personal attributes; e.g., lack of interest of knowledge), interpersonal (related to relations; e.g., lack of friends), and structural (related to situations; e.g., time, cost) constraints; efforts to overcome constraints initiate and persist based on intrinsic and extrinsic motives [18, 30]. In this framework, constraints pose negative effects on behavior, and FOMO as an extrinsic motive can facilitate the overcoming of intrapersonal and structural constraints (cf. interpersonal constraints may not apply for being incompatible with FOMO). That is, FOMO can be proposed as a moderator lifting intrapersonal and structural constraints in stimulating behavioral intention related to sport consumption.

Hodkinson highlighted that FOMO “call[s] on consumers to directly address their internal hesitancy, or resistance, to assent to an action” (p. 66) [17]. In the constraint-effects-mitigation model [31], Hubbard and Mannell examined leisure motives as direct positive stimulators of behavior and as mitigators (via negotiation) of constraint’s negative effect on behavior; the mitigator function was supported in their study and others [32, 33]. Such findings align with our proposal that FOMO as an extrinsic motive can be a factor facilitating the lifting of constraints. No study has empirically examined the moderating role of FOMO concerning sport consumption, calling for verification. Hypotheses 1, 2, and 3 are tested in the sport event context.

H1: FOMO is positively associated with behavioral intention.
H2a: Intrapersonal constraint is negatively associated with behavioral intention.
H2b: Structural constraint is negatively associated with behavioral intention.
H3a: The negative association between intrapersonal constraint and behavioral intention weakens as FOMO increases.
H3b: The negative association between structural constraint and behavioral intention weakens as FOMO increases.

### FOMO-driven consumption, rewards, and consumer satisfaction

FOMO can affect consumption experiences as well as decisions. The SDT posits that intrinsic and extrinsic motives prompt behavior, and their fulfillment determines the psychological experience associated with the behavior [13]. Intrinsic and extrinsic motives are fulfilled as intrinsic (i.e., enjoyment) and extrinsic rewards (e.g., social status, risk avoidance) are respectively attained. The type and strength of motives shape one’s objectives and expectations for rewards, consequently affecting motive fulfillment and thus the experience entailed.

As based on the extrinsic motive of avoiding risks of incompetence and lacking relatedness, the objectives and expectations of FOMO-driven behavior are mainly focused on the extrinsic reward of removing social apprehensions. In evidence, Kang et al. found FOMO-driven consumption to bring the extrinsic rewards of reinforced social stability and lessened social concern [6]. In sport management, FOMO-relevant motives such as social affiliation and knowledge acquisition are known to be predictors of sport consumption [7, 20, 27], insinuating that such extrinsic rewards can be expected from the consumption. Whereas FOMO-driven behavior mainly leads to extrinsic rewards such as approval and group integration, Argan and Argan noted that intrinsic rewards can also be gained via instant need gratification [19]. Social-pressure-driven behavior was found to draw enjoyment by fostering impressions of competence and relatedness [34]. FOMO-driven sport event consumption can be expected to mainly lead to extrinsic rewards but in part to intrinsic rewards as well. Thus, Hypothesis H4 is proposed.

H4a: FOMO-driven sport event consumption is associated with intrinsic rewards.
H4b: FOMO-driven sport event consumption is associated with extrinsic rewards.
H4c: FOMO-driven sport event consumption’s association with extrinsic rewards is stronger than that with intrinsic rewards.

While FOMO-driven consumption can derive intrinsic and extrinsic rewards, the relation between the rewards requires attention. FOMO was introduced as an extrinsic motive that socially compels and enforces behavior. In the SDT (referring to the mini-theory of cognitive evaluation) [13], extrinsic rewards acquired at the expense of autonomy are known, in part, to undermine intrinsic motives/rewards. The relation was demonstrated with extrinsic rewards such as avoiding threats and penalties impeding pure enjoyment [13, 35]. Bartholomew, Ntoumanis, and Thogersen-Ntoumanis discussed controlling-strategies of coaches (e.g., competition, surveillance) to coerce players to strive for extrinsic rewards (e.g., more attention, less scolding), which attentiveness to obtained extrinsic rewards thwarts intrinsic rewards [36]. There are shreds of evidence for extrinsic rewards from FOMO-driven behavior oftentimes entailing regret, stress, and negative moods [1, 37]; such negative states contrast intrinsic rewards and are generated when extrinsic rewards come with mental burdens and/or opportunity costs [17]. The relation between extrinsic and intrinsic rewards can be anticipated in FOMO-driven sport event consumption, leading to Hypothesis 5.

H5: Extrinsic rewards are negatively associated with intrinsic rewards.

As noted in the SDT, intrinsic and extrinsic rewards lead to satisfactory experiences [22]. The positive influence of reward attainment (or motive fulfillment) on the psychological experiences of sport event consumers has been well documented [25, 38]. Intrinsic rewards are known to be stronger predictors of satisfactory experiences than extrinsic rewards, as (1) pure enjoyment is more directly linked to inherent satisfaction than instrumental outcomes, and (2) extrinsic rewards are more restricted in satisfying autonomy need [13]. Intrinsic and extrinsic rewards’ strength of influence on consumer satisfactory experiences has not been assessed and compared in the sport consumption context. Hypothesis 6 is tested with sport event consumers.

H6a: Intrinsic rewards are associated with consumer satisfaction.
H6b: Extrinsic rewards are associated with consumer satisfaction.
H6c: Intrinsic reward’s association with consumer satisfaction is stronger than that with extrinsic rewards.

With Hypotheses 4, 5, and 6 altogether, extrinsic rewards are projected to be more strongly associated with FOMO-driven consumption and negatively linked to intrinsic rewards. However, intrinsic rewards can be more important for consumer satisfaction. If this is the case, FOMO-driven consumption’s impact on consumer satisfaction may be questionable, calling for attention. Testing the hypotheses will provide valuable insights on utilizing FOMO in sport marketing strategies.

## Method

**Fig 1:**
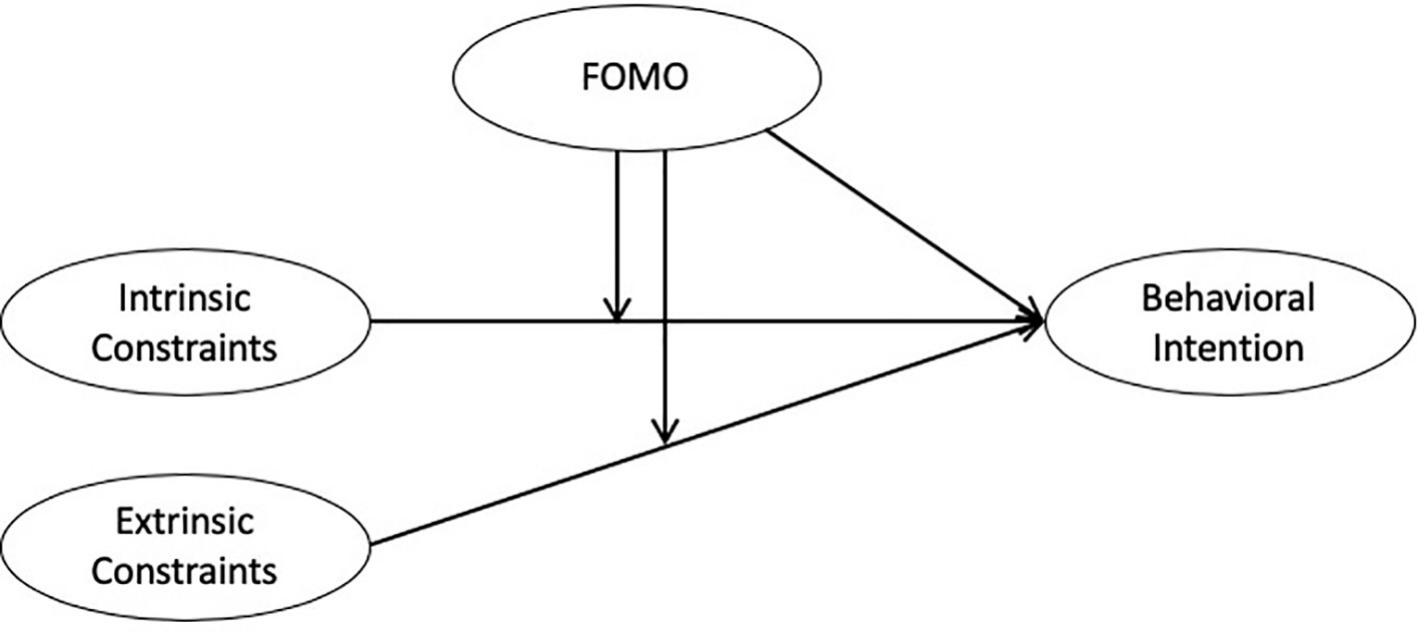
Structural Model of Study 1.

**Fig 2:**
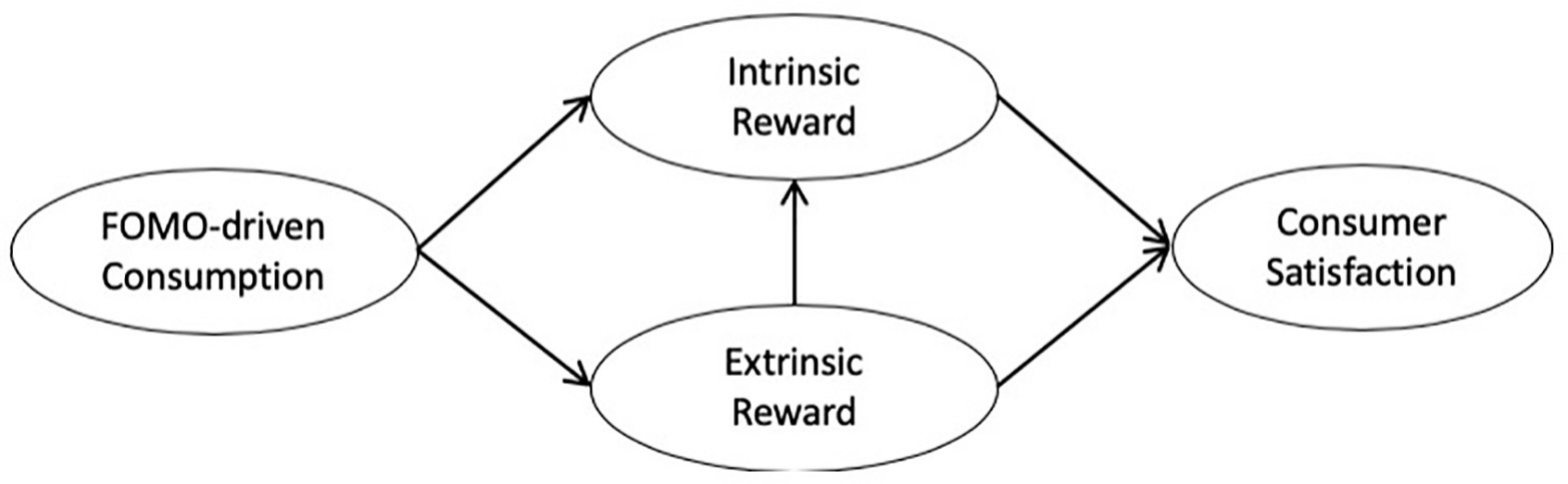
Structural Model of Study 2.

Two online survey-based studies were conducted. Study 1 examined FOMO as an extrinsic motive and moderator weakening the negative link between constraints and sport media viewing intention (H1~3; Fig 1). Study 2 assessed how FOMO-driven sport event consumption is associated with intrinsic/extrinsic rewards and consumer satisfaction (H4~6; Fig 2). The Super Bowl was selected as the research context since it is (1) the most popular sport event in the United States in terms of viewership that (2) many perceive as ‘the one big game not to miss’ [8].

### Participants and sampling

The target population of Study 1 was U.S. adults who intended to watch Super Bowl 2020 through media (i.e., TV or Internet). As the purpose of this study was to understand FOMO as an extrinsic motive for sport event consumption, it was critical to capture one’s FOMO and intention specifically related to the sport event content. Thus, we limited our focus to those planning to watch Super Bowl 2020 on their own; we excluded those planning to watch at social gatherings (e.g., party) from the study, as FOMO and intention in such case may be formed or affected by the social gathering rather than the sport event content. Participants were recruited one day before the Super Bowl 2020 using Amazon Mechanical Turk (i.e., “mTurk”) [39]. The time was selected to capture FOMO after being exposed to the popularity of and hype before the Super Bowl 2020 and after intention for watching the event had been more concretely formed [7]. Two screening questions (i.e., plan to watch the event, plan to watch at social gatherings) and convenience quota sampling (based on demographics) were used in the recruitment process.

For Study 2, the target population was U.S. adults who watched Super Bowl 2020 through media but not at social gatherings. Participants were recruited after the event to accurately measure intrinsic/extrinsic rewards and satisfaction related to the event experience; the recruitment was based on screening questions (i.e., watched the event, watched at social gatherings), convenience quota sampling (based on demographics), and mTurk.

### Instruments

Items to measure FOMO focused on capturing one’s perceived level of Super-Bowl-driven FOMO at the time of data collection (regardless of whether FOMO extrinsically stemmed from external stimulation or internalized self-regulation) and were adopted and revised from two existing scales. First, Przybylski and colleagues’ scale was chosen for its popularity and theoretical basis in SDT [1]. Second, Wegmann and colleagues’ scale was selected for its applicability to trait- and state-FOMO [24] and for being developed based on Przybylski and colleagues’ scale [1]. Both scales have been commonly used and reported strong psychometric properties in studies [2, 6, 15]. Six items were selected and modified to fit our context, developed into 7-point Likert-type scales, and reviewed by experts to ensure face and content validity. A pilot test was conducted with college students (*n* = 72) to check reliability and validity (e.g., inter-item correlation, composite reliability, factor loading) [40]. The items were deemed adequate for use. Details of items are presented in Table 1.

**Table 1:** Study 1: Psychometric properties.

Dimensions and Scale Items (n = 219)	Est.	S.E.	Est./S.E.	AVE	α	ρ	Mean	SD
FOMO*				.77	.95	.95		
1. I fear missing out on a rewarding experience.	.73	.04	18.58				2.99	1.79
2. I fear others may have a rewarding experience without me.	.89	.02	42.30				3.78	1.94
3. It is important to know about an event that others will be experiencing.	.90	.02	41.21				3.81	1.88
4. It is important to be part of an event that others will be experiencing.	.90	.02	38.00				4.07	1.90
5. I fear not being up-to-date on an event that others will be experiencing.	.89	.03	32.90				3.56	1.85
6. I get anxious when I miss out on an event that others will be experiencing.	.93	.01	75.66				3.70	1.87
**Intrapersonal Constraint (Lack of Interest)**				.57	.87	.86		
1. I am not interested in the Super Bowl.	.78	.04	22.11				3.10	1.41
2. I am not interested in American football.	.66	.05	12.40				2.84	1.38
3. I am not interested in the Super Bowl halftime show.	.53	.06	8.33				3.29	1.35
4. I am not interested in the Super Bowl commercials.	.91	.03	32.46				2.83	1.35
5. I am not interested in the two teams competing in the Super Bowl.	.84	.03	26.70				2.82	1.44
**Structural Constraint (Other Commitments)**				.78	.91	.91		
1. I cannot watch the Super Bowl due to work or study commitments.	.96	.02	39.83				2.28	1.54
2. I cannot watch the Super Bowl due to social or family commitments.	.86	.04	22.98				2.27	1.70
3. I cannot watch the Super Bowl due to other commitments.	.82	.03	24.52				2.15	1.51
**Behavioral Intention**				.92	.96	.97		
1. I am planning to watch the Super Bowl.	.97	.01	95.56				5.80	1.73
2. I intend to watch the Super Bowl.	.96	.01	73.78				5.85	1.74
3. The possibility of me watching the Super Bowl is [very low-very high].	.96	.02	59.14				5.86	1.71

* Items were led by [I am planning to watch the Super Bowl because…].

After reviewing constraints literature and conducting expert panel discussions [7, 20, 41, 42, 43], ‘lack of interest’ and ‘other commitments’ were respectively selected as key intrapersonal and structural constraints relevant to our research context. Items to measure the two constraints were constructed. While we acknowledge that the two constraints may not be exhaustive, focusing on the two as representative examples of intrapersonal and structural constraints was deemed sufficient to serve our primary purpose of demonstrating FOMO’s moderation between constraint and intention. Based on Alexandris and Stodolska’s item (i.e., ‘I am not interested’) [41], five items to measure ‘lack of interest’ were developed, each touching on key sources of interest in the Super Bowl viewing experience (e.g., event, sport, teams, commercials, halftime show; identified based on Apostolopoulou, Clark, and Gladden) [44]. Three items to assess ‘other commitments’ were employed from Trail and Kim [43]. All items were 7-point Likert-type scales and self-reported based on subjectively perceived levels of constraints (Table 1).

Intrinsic rewards were assessed with intrinsic motivation items; the items were adopted from Guay, Vallerand, and Blanchard’s situational motivation scale that is popularly used and known to have strong psychometric properties [45]. As the intrinsic motivation items capture one’s expectations for intrinsic rewards from an activity (e.g., “because this activity is fun”) [45], the items were reworded to assess one’s experience of intrinsic rewards in watching the Super Bowl (e.g., “watching the Super Bowl was fun”). Five items were employed to measure the subjectively perceived and recalled experience of intrinsic rewards. Extrinsic rewards items came mainly from social adherence items in Martela, Bradshaw, and Ryan’s extrinsic aspiration scale [46]. Three items were revised to fit the Super Bowl context and one item was constructed; the items captured subjectively perceived extrinsic rewards based on recall. Details of items measuring intrinsic and extrinsic rewards are shown in Table 2 (cf. all in 7-point Likert-type scales).

**Table 2:** Study 2: Psychometric properties.

Dimensions and Scale Items (n = 255)	Est.	S.E.	Est./S.E.	AVE	α	ρ	Mean	SD
FOMO*				.66	.92	.92		
1. I fear missing out on a rewarding experience.	.78	.03	26.53				3.78	1.84
2. I fear others may have a rewarding experience without me.	.69	.04	17.25				3.22	1.78
3. It is important to know about an event that others will be experiencing.	.86	.02	36.09				4.14	1.85
4. It is important to be part of an event that others will be experiencing.	.83	.02	36.07				4.00	1.82
5. I fear not being up-to-date on an event that others will be experiencing.	.84	.03	28.78				4.47	1.77
6. I get anxious when I miss out on an event that others will be experiencing.	.84	.03	27.08				4.31	1.77
**Intrinsic Reward**				.61	.89	.89		
1. Watching the Super Bowl was interesting.	.76	.05	16.36				5.44	1.27
2. Watching the Super Bowl was pleasant.	.80	.04	19.48				5.49	1.29
3. Watching the Super Bowl was fun.	.81	.03	23.50				5.63	1.18
4. I felt good after watching the Super Bowl.	.78	.04	19.01				5.35	1.29
5. I felt excited after watching the Super Bowl.	.76	.04	18.21				5.06	1.45
Extrinsic Reward**				.77	.93	.93		
1. I felt more accepted by my peers.	.92	.02	60.14				3.45	1.68
2. I felt more approved of by the people around me.	.90	.02	43.80				3.40	1.67
3. I felt more included in social circles.	.89	.02	42.89				3.47	1.63
4. I fit in better with others.	.78	.03	23.26				3.67	1.72
**Consumer Satisfaction**				.70	.91	.90		
1. I am very satisfied with my experience watching the Super Bowl.	.85	.03	27.84				5.31	1.33
2. My choice to watch the Super Bowl was a wise one.	.80	.03	25.17				5.21	1.35
3. Really, I have enjoyed watching the Super Bowl.	.90	.03	33.96				5.25	1.38
4. I don’t regret watching the Super Bowl at all.	.80	.05	14.99				5.54	1.29

* Items were led by [I watched the Super Bowl because…].

** Items were led by [After watching the Super Bowl…].

Three items to measure one’s intention to watch the Super Bowl were constructed based on Cronin, Brady, and Hult’s [47] and Cunningham and Kwon’s [48] studies; two items were 7-point Likert-type scales and one was a 7-point semantic differential scale. Four items for consumer satisfaction were from Caro and Garcia’s study (7-point Likert-type scales) and were answered based on subjective self-reports and recall about the Super Bowl viewing experience [49]. Five items for demographics (i.e., gender, age, race, education, income) were developed. For control purpose (i.e., to eliminate the influence of game content/outcome on intrinsic/extrinsic rewards and consumer satisfaction in Study 2), one item to assess performance satisfaction was constructed (“the performance of the team I supported was [very dissatisfactory-very satisfactory]”; 7-point).

Two questionnaires were designed for online surveys. The questionnaire for Study 1 included items for FOMO (6 items), intrapersonal constraint (5 items), structural constraint (3 items), intention (3 items), and demographics (5 items). In the questionnaire for Study 2, items for FOMO-driven consumption (6 items), intrinsic rewards (5 items), extrinsic rewards (4 items), consumer satisfaction (4 items), performance satisfaction (1 item), and demographics (5 items) were included. Following Podsakoff, MacKenzie, and Podsakoff’s guideline, proximal separation, elimination of ambiguous words, request for candid responses, assurance of anonymity, and adoption of different scale-types were applied in the survey design to minimize common method variance issues [50]. After a final round of expert review, the two questionnaires were considered ready for use.

### Data collection and analysis

Data were collected after obtaining the approval from the Institutional Review Board (IRB) at Syracuse University for this study (approval number: 20–021). For Study 1, data were collected one-day before the Super Bowl 2020 based on convenience quota sampling via mTurk (cf. $.25 credit offered per respondent); electronic written consents were obtained from respondents. While mTurk is known as a useful platform for recruiting a diverse sample of survey respondents in an inexpensive way, concerns of deceptive responses and representativeness needed to be considered [39]. To address such concerns, (1) only mTurk workers with more than 95% HIT approvals were recruited, (2) several screening questions were included to ensure the respondents matched our eligibility criteria, and (3) demographic characteristics of respondents were compared to that of U.S. adult NFL consumers and the U.S. general population.

As a result, 219 usable responses were collected after excluding 23 incomplete (i.e., listwise deletion) and unreliable responses (e.g., all answers were identical). Demographic characteristics of respondents were as follow: [gender: 52.1% male; average age: 44.8; race: 72.1% white; median household income: ranging between $50,000 and 100,000; education: 50.2% with 4-year or post-graduate degree]. The demographics of our respondents fairly matched that of U.S. adult NFL consumers and U.S. general population, except for education. The demographics of U.S. adult NFL consumers were [gender: 58% male; average age: 48.9; race: 77% white; education: 34% with 4-year or post graduate degree; household income: 27% with $75,000+] [51, 52] and that of U.S. general population were [gender: 49.2% male; average age: 38.2 (non-adults included in estimation); race: 76.3% white; education: 31.5% with 4-year or post graduate degree; median household income: $60,293] [53]. Using *MPlus7*, data analysis was conducted with Structural Equation Modeling with Moderation (cf. based on Hayes’ PROCESS Model 1 and Stride, Gardner, Catley, and Thomas’ syntax to test Hayes’ Model 1 with latent variables) [54, 55]; FOMO’s moderation between (1) intrapersonal constraint and intention and (2) structural constraint and intention were tested in two separate models.

For Study 2, data collection took place after the Super Bowl 2020 with convenience quota sampling and mTurk (cf. $.25 credit paid per respondent); electronic written consents were attained. Excluding 34 incomplete and unreliable responses, 255 responses were usable. Respondent’s demographic information were [gender: 57.2% male; average age: 47.9; race: 80.0% white; median household income range: $50,000~100,000; education: 54.9% has 4-year or post-graduate degree], similar to that of Study 1. Data were analyzed to test the associations among FOMO-driven consumption, intrinsic and extrinsic rewards, and consumer satisfaction based on Structural Equation Modeling and *MPlus7*. Performance satisfaction was controlled (as a covariate) for intrinsic and extrinsic rewards and consumer satisfaction to cancel the unwanted influence of game content/outcome [25].

## Results

### Study 1

The measurement model with FOMO, intrapersonal and structural constraints, and intention scales was tested. While data satisfied the assumptions of missing data, normality, linearity, and singularity, a violation of multivariate normality was reported (Mardia’s coefficient of kurtosis at *p* < .01) [56]. Satorra-Bentler correction was employed [57]. Confirmatory Factor Analysis was conducted. Measurement model fit was satisfactory (S-B χ2/df = 256.648/113 = 2.27, CFI = .94, TLI = .93, SRMR = .06, RMSEA = .08, and 90% C.I. [.06-.09]) based on Hu and Bentler’s criteria [58]. Cronbach’s alpha ranged from .87 to .96 and composite reliability from .86 to .97; all values were greater than .70 (Table 1) [59]. Factor loadings ranged from .53 to .97; all values were greater than .70 in predicted directions [60] with two exceptions. AVE values ranged from .57 to .92, with all values greater than .50 and the respective squared inter-factor correlations (Table 3) [61]. Multiple χ2 difference tests of unity were performed for all pairs of constructs. Results reported significantly better fits in unconstrainted models than in constrained models in all pairs (all ΔS-B χ2 at *p* < .01). Overall, the measurement model had good reliability, convergent validity, and discriminant validity.

**Table 3:** Inter-factor correlations, means, and standard deviations.

Study 1	Factors		1.	2.	3.	Construct Mean
	1.	FOMO				3.65
	2.	Intrapersonal Constraint	-.01			3.08
	3.	Structural Constraint	.07	.05		2.28
	4.	Behavioral Intention	.35	-.28	-.40	5.84
Study 2	Factors		1.	2.	3.	Construct Mean
	1.	FOMO				3.98
	2.	Intrinsic Reward	.17			5.39
	3.	Extrinsic Reward	.50	-.02		3.50
	4.	Consumer Satisfaction	.25	.67	.15	5.33

Two structural models were examined (Table 4). In the first model, the relations among FOMO, intrapersonal constraint, and intention were tested. Structural Model fit was good (S-B χ2/df = 198.961/74 = 2.69, CFI = .94, TLI = .93, SRMR = .06, RMSEA = .08, and 90% C.I. [.07-.09]. Paths from FOMO to intention (γ = .52, S.E. = .10, p < .01) and from intrapersonal constraint to intention (γ = -.39, S.E. = .10, p < .01) were significant in the predicted directions. A significant moderation effect of FOMO on the path from intrapersonal constraint to intention was reported (γ = .24, S.E. = .09, p < .01). The analyses also provided an assessment of conditional effects among those with low (- 1 SD), medium (mean), and high (+1 SD) levels of FOMO [54, 55]. The strength of association between intrapersonal constraint and intention varied among those who experienced low (γ = -.62, S.E. = .14, p < .01), medium (γ = -.39, S.E. = .10, p < .01), and high (γ = -.15, S.E. = .13, p = .24) levels of FOMO. H1, H2a, and H3a were supported.

**Table 4:** Study 1: Path coefficients of the Structural Models.

Path Coefficients	Est.	S.E.	Est./S.E.	*p*-value
**Model 1**				
FOMO → Behavioral Intention	.52	.10	-3.81	< .01
Intrapersonal Constraint → Behavioral Intention	-.39	.10	5.09	< .01
FOMO x Intrapersonal Constraint → Behavioral Intention	.24	.09	2.66	< .01
• Conditional Effects -				
: of Intrapersonal Constraint → Behavioral Intention				
Low FOMO (-1 SD)	-.62	.14	-4.47	< .01
Medium FOMO (M)	-.39	.10	-3.81	< .01
High FOMO (+1 SD)	-.15	.13	-1.19	.24
**Model 2**				
FOMO → Behavioral Intention	.61	.10	-7.21	< .01
Structural Constraint → Behavioral Intention	-.53	.07	6.40	< .01
FOMO x Structural Constraint → Behavioral Intention	.24	.07	3.70	< .01
• Conditional Effects -				
: of Structural Constraint → Behavioral Intention				
Low FOMO (-1 SD)	-.77	.11	-7.12	< .01
Medium FOMO (M)	-.53	.07	-7.21	< .01
High FOMO (+1 SD)	-.29	.09	-3.31	< .01

The relations among FOMO, structural constraint, and intention were tested in the second model. Structural Model Fit was adequate (S-B χ2/df = 100.783/51 = 1.98, CFI = .97, TLI = .96, SRMR = .07, RMSEA = .07, and 90% C.I. [.05-.09]). FOMO (γ = .61, S.E. = .10, p < .01) was positively and structural constraint was negatively (γ = -.53, S.E. = .07, p < .01) associated with intention. The moderation of FOMO on the path from structural constraint to intention was significant (γ = .24, S.E. = .07, p < .01). The strength of association between structural constraint and intention differed among those reporting low (γ = -.77, S.E. = .11, p < .01), medium (γ = -.53, S.E. = .07, p < .01), and high (γ = -.29, S.E. = .09, p < .01) levels of FOMO. H1, H2b and H3b were all supported.

### Study 2

The measurement model of FOMO, intrinsic/extrinsic rewards, and consumer satisfaction scales was tested. After checking for key assumptions (e.g., missing data, normality, linearity, singularity), a concern about multivariate normality was raised as Mardia’s coefficient of kurtosis was significant (*p* < .01) [56]; Satorra-Bentler correction was adopted [57]. Good fit was reported for the Measurement Model (S-B χ2/df = 299.784/146 = 2.05, CFI = .93, TLI = .92, SRMR = .06, RMSEA = .06, and 90% C.I. [.05-.08]) based on Hu and Bentler’s criteria [58]. All values of Cronbach’s alpha (from .89 to.93) and composite reliability (from .89 to .93) were greater than .70 (Table 2) [59]. All factor loadings (from .69 to .92) were greater than .70 and in predicted directions [60], except one. All AVE values (from .61 to .77) were greater than .50 and the relevant squared inter-factor correlations (Table 3) [61]. Results of multiple χ2 difference tests of unity for all pairs of constructs showed better fits in unconstrained models than in constrained models (all ΔS-B χ2 at *p* < .01). Satisfactory reliability, convergent validity, and discriminant validity of the measurement model were demonstrated.

Structural Equation Modeling was conducted to examine the relations among FOMO-driven consumption, intrinsic and extrinsic rewards, and consumer satisfaction (Table 5) with performance satisfaction controlled. Structural Model Fit was adequate (S-B χ2/df = 335.363/163 = 2.06, CFI = .93, TLI = .92, SRMR = .06, RMSEA = .06, and 90% C.I. [.06-.07]). Paths from FOMO to intrinsic reward (γ = .24, S.E. = .09, p < .01; 95% C.I. [.11-.38]) and extrinsic reward (γ = .51, S.E. = .06, p < .01; 95% C.I. [.40-.61]) were both significant; the valence (or coefficients) of the two paths were statistically different, as the 95% C.I. of each path coefficient did not overlap [62]. Extrinsic reward was marginally significantly and negatively associated with intrinsic reward (γ = -.13, S.E. = .08, p = .10). Intrinsic reward (γ = .69, S.E. = .08, p < .01; 95% C.I. [.54-.82]) and extrinsic reward (γ = .16, S.E. = .07, p < .02; 95% C.I. [.05-.27]) were both positively linked to consumer satisfaction; there was no overlap between the two paths’ 95% C.I. of coefficients, indicating that intrinsic reward’s association with consumer satisfaction was statistically stronger than that with extrinsic reward. Overall, H4, H5, and H6 were supported. The total and indirect effect from FOMO to consumer satisfaction was γ = .20 (S.E. = .05, p < .01), explaining 4% of satisfaction’s variance. The indirect effect through the mediation of intrinsic reward was γ = .17 (S.E. = .07, p = .01), through the mediation of extrinsic reward was γ = .08 (S.E. = .04, p = .03), and through the mediation of extrinsic and then intrinsic rewards was γ = -.05 (S.E. = .03, p = .11).

**Table 5:** Study 2: Path coefficients of the Structural Model.

Path Coefficients	Est.	S.E.	Est./S.E.	*p*-value
FOMO → Intrinsic Reward	.24	.09	2.88	< .01
FOMO → Extrinsic Reward	.51	.06	8.08	< .01
Extrinsic Reward → Intrinsic Reward	-.13	.08	-1.67	.10
Intrinsic Reward → Consumer Satisfaction	.68	.08	8.17	< .01
Extrinsic Reward → Consumer Satisfaction	.16	.07	2.37	.02

## Discussion

The contribution of this project lies in (1) bringing attention to FOMO as a psychological condition affecting decision-making for and experience in sport event consumption; (2) providing theoretical accounts and empirical pieces of evidence supporting the role of FOMO in shaping consumer behavior as a stimulation for intention and a moderator lifting constraints; and (3) verifying FOMO-driven behavior to be mainly grounded on extrinsic rewards whereas intrinsic rewards are the prevailing predictor of consumer satisfaction. The findings indicate FOMO as a meaningful extrinsic motive for sport event consumption but question its effects on the consumer’s satisfying experience. The results offer novel insights into how FOMO should be approached for consumer acquisition and retention.

### Study 1

The relationship between FOMO and sport event consumption intention can be explained through two distinctive mechanisms. First, FOMO was positively associated with intention, directly explaining 27.0% and 37.2% of intention’s variance in Models 1 and 2. The finding adds to the body of research identifying FOMO as a meaningful motive of behavior formed based on concerns of incompetence and lack of relatedness [6, 14, 19] and social apprehension as a trigger for sport consumption [9, 11, 12]. As an extrinsic motive, FOMO’s association with sport event consumption intention was strong, which was somewhat inconsistent with previous studies reporting modest or insignificant effects of extrinsic motives (e.g., promotional deals, advertisements) [25, 63]. Such finding can be explained in that FOMO can be distinctive from other extrinsic motives for being based on ‘loss aversion’ (or risk avoidance) (i.e., people react more sensitively to potential losses than gains) [64], which loss comes from missing out on opportunities to fulfill other key motives (e.g., social affiliation, vicarious achievement, knowledge acquisition) [20]. That is, grounded on fear and that fear encompassing other key motives, FOMO can function as an important predictor for sport event consumption intention.

Second, the negative association between constraint and sport event consumption intention weakened as one’s perceived level of FOMO increased, demonstrating FOMO’s moderation effect; the interaction effects between FOMO and constraints accounted for 5.8% of intention’s variance in each model. While the moderating role of motive has been hinted in leisure studies [31, 32, 33], this project is perhaps one of the first in the sport field to empirically examine the role with FOMO. The mechanism brings the interaction of constraints and motives into the scope of understanding intention formation, providing insights on the initiation and persistence of efforts in overcoming constraints [30]. The finding supports Hodkinson’s argument that FOMO can effectively coerce individuals to deal with their hesitancy in taking an action [17]. Notably, while intrapersonal and structural constraints respectively explained 15.2% and 28.1% of intention’s variance, FOMO perceived among the high group was strong enough to cancel the association between intrapersonal constraint and intention (p = .24) and diminish that of structural constraint to explaining 8.4% of intention’s variance (γ = -.29, p < .01). These findings support the effectiveness of FOMO in facilitating the process of surmounting both constraint-types and suggest intrapersonal constraints to be easier to deal with in this context, perhaps as formed based on internal factors that are fully and mentally controllable by self and not affected by uncontrollable external/structural factors [29].

As a motive stemming from concerns about incompetence and lack of relatedness caused by missing out on popular and/or satisfying experiences [6, 14, 19], FOMO was verified as a meaningful factor in the intention formation for sport event consumption. The motive can be effective and unique for being based on risk avoidance, encompassing other key motives, imposing external coercion, and facilitating the lifting of constraints [15, 17]. The foundation of FOMO is more reliant on extrinsic forces, allowing marketers easier and immediate manipulation than intrinsic forces/motives. Such an aspect enhances the functionality of FOMO in the intention formation process, calling for further research.

### Study 2

FOMO-driven consumption was positively linked to both reward-types, explaining 26.0% of extrinsic rewards’ and 5.8% of intrinsic rewards’ variances. The stronger FOMO was perceived in the decision-making, the more extrinsic rewards were reported in consumption, as FOMO-driven consumers were likely to be more committed and attentive to instrumental social gains that could relieve concerns of incompetence and lack of relatedness [6]. FOMO-driven consumption was also linked to intrinsic rewards, which rewards occur when pleasure is experienced through genuine satisfaction of psychological needs [13]; in this case, competence and relatedness needs [34]. That is, while primarily instrumental, social gains in FOMO-driven consumption may have in part led to a sincere appreciation of benefits such as knowledge acquisition and social affiliation [19]. Overall, extrinsic social rewards were confirmed as the more prominent benefit associated with FOMO-driven consumption.

Sport consumer satisfaction was better explained by intrinsic rewards (46.2%) than extrinsic rewards (2.6%), as anticipated based on SDT [13, 22]. Extrinsic rewards had weaker associations with consumer satisfaction than intrinsic rewards, consistent to studies reporting intrinsic factors as stronger motives for sport event consumption [20, 63] and motive fulfillment as a key determinant of the consumer’s positive experience [25, 38]. The finding points out intrinsic rewards as the more crucial construct in determining consumer satisfaction.

The extrinsic rewards of FOMO-driven consumption reported a marginal but negative (γ = -.13, p = .10) relation to intrinsic rewards. The finding supports the Cognitive Evaluation Theory in SDT [13], which explains such relation with extrinsic rewards being associated with extrinsic influence/coercion that disrupts autonomy need satisfaction and thereby intrinsic rewards. FOMO-driven consumption occurs out of risk/threat avoidance, which is an extrinsic reward-type pursued under the pressure of risk/threat and therefore at the expense of autonomy [65]. Attentiveness to extrinsic rewards can interrupt the pure enjoyment of the experience as well, thwarting intrinsic rewards [36].

Overall, FOMO-driven consumption indirectly explained a rather small 4% of consumer satisfaction’s variance. FOMO-driven consumption was more closely linked to extrinsic rewards despite intrinsic rewards being the stronger predictor of consumer satisfaction, and the extrinsic rewards had a marginal but negative association with intrinsic rewards. That is, FOMO may be useful for prompting behavioral intention, but FOMO-driven consumption may face challenges in satisfying and retaining consumers. The findings are meaningful for raising concerns on the value of FOMO-driven marketing in establishing long-term and voluntary relationships with sport consumers and for understanding intrinsic and extrinsic rewards under conditions of extrinsically coerced consumption.

### Practical implications

With easier manipulation as an extrinsic/external factor and effective leveraging based on two mechanisms, FOMO-driven marketing can be suggested as a useful strategy for stimulating sport event consumption intention. The extrinsic motive can act as an alternative or supplement to intrinsic motives in prompting behavioral intention, especially helpful for acquiring consumers who initially lack intrinsic interest. As popularity within social groups is a prerequisite for FOMO [14], selective targeting of events and marketing activities geared toward relevant social groups are required (e.g., NCAA event at college campuses, Olympics in the host country, sport camps for the youth). To simulate FOMO, positioning a sport event as a popular social trend (e.g., by involving influencers, enhancing media presence, using buzz marketing) and priming consumers to think about the consequences of missing out (e.g., being non-conversant, being left out in shared memory) can be effective. Targeting such efforts at the time when consumers face constraints (e.g., paying for tickets) and toward individuals dealing with constraints (e.g., lacking interest, busy schedule) can be a way to stimulate intentions by assisting consumers in surmounting constraints with FOMO. FOMO-appeal can be more compelling among younger generations, in social media space, and when raised by meaningful others [17]. One point to note is that excessive stimulation of fear can threaten consumers’ well-being [1, 37], calling for caution in FOMO-driven marketing.

While FOMO can be effective for boosting intention, FOMO-driven consumption has limitations in enhancing consumer satisfaction and retention, for being closely linked to extrinsic rewards although intrinsic rewards are more influential on satisfaction and for being conditional based on FOMO unlike voluntary consumption made for intrinsic rewards. Strategies to better connect FOMO-driven consumption to intrinsic rewards need to be accompanied for increased consumer satisfaction and retention. For this, ensuring consumers to obtain and recognize intrinsic rewards (i.e., pleasure) and other non-instrumental psychological benefits (e.g., escape, drama, aesthetics, eustress) over the course of FOMO-driven consumption are important.

Another suggestion is to convert the marginal but negative link between FOMO-driven extrinsic and intrinsic rewards into a positive relation. In the Cognitive Evaluation Theory in SDT [13], there is a specified limiting condition for the negative link that extrinsic rewards provided in the form of positive feedback do not undermine intrinsic rewards (cf. confirmed in a meta-analysis) [65]. Such a condition suggests providing positive feedback alongside extrinsic social rewards as a useful way to link FOMO-driven consumption more effectively to intrinsic rewards (e.g., acknowledging social gains and recognizing social circles).

### Limitation and future directions

Whereas valuable and novel insights on the effectiveness and uniqueness of FOMO as a motive (e.g., risk avoidance, extrinsic coercion, lifting constraints) and concerns in associated experience (e.g., mixed effects on intrinsic rewards) were obtained through this study, further research on FOMO is needed. First, delving into the trait-state distinction of FOMO is suggested. While FOMO can be approached as a trait and/or a state [24, 66], the construct was simply captured based on one’s perceived level of FOMO at the time of data collection in this study. The approach well-served the purpose of this research. However, further trait-state specification of FOMO based on whether spurred by internalized self-regulation for social compliance or external stimulation can be informative (grounded on the Organismic Integration Theory in SDT) [13]. Such specification can provide insights on the duration of FOMO, ways to stimulate FOMO, and vulnerability to FOMO-driven marketing. Knowledge of trait- and state-like FOMO in sport event consumption can be distinctively applied to strategies for sport consumer retention and acquisition.

Second, the identification of factors influencing the stimulation of FOMO is required. FOMO acts upon concerns about incompetence and lack of relatedness, which concerns are engendered from bypassing opportunities to satisfy key motives. Identifying which concern-type or motive-type is more effective in prompting FOMO can inform FOMO-driven marketing strategies. As FOMO is grounded on social apprehension, testing factors such as event popularity, size and homogeneity of a social group, collectivism-based culture, FOMO-stimulating agents, SNS usage, self-construal, and personality traits can avail knowledge on the antecedents of FOMO [12, 15, 17, 18]. Examining how FOMO can be amplified when a popular sport event is combined with a social gathering (e.g., watch party, tailgating) can also provide valuable insights.

Lastly, further research on the link between intrinsic and extrinsic rewards associated with FOMO is necessary. Earlier, it was suggested that, whereas extrinsic rewards are generally negatively linked to intrinsic rewards, extrinsic rewards provided in a form of positive feedback may increase intrinsic rewards [13, 65]; verification in the sport event consumption context is required, perhaps through experimental research design. As previous studies report that mental states caused by FOMO-driven behavior can range from positive (e.g., enjoyment, self-esteem, and relief) to negative (e.g., stress, guilt, and regret) in which these states are known to affect intrinsic rewards [19, 37], incorporating FOMO-driven mental states into the research scope and investigating the conditions that determine the valence of each state is recommended.

## Supporting information

S1 File Data set of Study 1. (CSV)

S2 File Data set of Study 2. (CSV)

S3 File Survey items. (DOCX)
