# Supplementary material for: Investigating ‘Fear of Missing Out’ (FOMO) as an extrinsic motive affecting sport event consumer’s behavioral intention and FOMO-driven consumption’s influence on intrinsic rewards, extrinsic rewards, and consumer satisfaction
Source: PLoS One. 2020 Dec 14;15(12):e0243744. doi: 10.1371/journal.pone.0243744 (PMC7735608; doi:10.1371/journal.pone.0243744)
Supplement: S3 File — (DOCX) [file pone.0243744.s003.docx]

**Survey Items for Study 1**

**Screening Questions (2 items)**

- Do you plan to watch the Super Bowl 2020? (Y/N)

- Do you plan to watch the Super Bowl 2020 at a social gathering? (Y/N)

**FOMO (6 items)**

I am planning to watch the Super Bowl because…

- I fear missing out on a rewarding experience.

- I fear others may have a rewarding experience without me.

- It is important to know about an event that others will be experiencing.

- It is important to be part of an event that others will be experiencing.

- I fear not being up to date on an event that others will be experiencing.

- I get anxious when I miss out on an event that others will be experiencing.

* 7-point Liker-type scales; anchors: 1 = strongly disagree, 7 = strongly agree.

**Intrapersonal Constraint (5 items)**

- I am not interested in the Super Bowl.

- I am not interested in American Football.

- I am not interested in the Super Bowl halftime show.

- I am not interested in the Super Bowl commercials.

- I am not interested in the two teams competing in the Super Bowl.

* 7-point Liker-type scales; anchors: 1 = strongly disagree, 7 = strongly agree.

**Structural Constraint (3 items)**

- I cannot watch the Super Bowl due to work or study commitments.

- I cannot watch the Super Bowl due to social or family commitments.

- I cannot watch the Super Bowl due to other commitments.

* 7-point Liker-type scales; anchors: 1 = strongly disagree, 7 = strongly agree.

**Behavioral Intention (3 items)**

- I am planning to watch the Super Bowl.

- I intend to watch the Super Bowl.

- The possibility of me watching the Super Bowl is [very low – very high]

* Items 1 & 2 were 7-point Liker-type scales; anchors: 1 = strongly disagree, 7 = strongly agree.

** Item 2 was a 7-point semantic differential scale.

**Survey Items for Study 2**

**Screening Questions (2 items)**

- Did you watch the Super Bowl 2020? (Y/N)

- Did you watch the Super Bowl 2020 at a social gathering? (Y/N)

**FOMO-driven Consumption (6 items)**

I watched the Super Bowl because…

- I fear missing out on a rewarding experience.

- I fear others may have a rewarding experience without me.

- It is important to know about an event that others will be experiencing.

- It is important to be part of an event that others will be experiencing.

- I fear not being up to date on an event that others will be experiencing.

- I get anxious when I miss out on an event that others will be experiencing.

* 7-point Liker-type scales; anchors: 1 = strongly disagree, 7 = strongly agree.

**Intrinsic Rewards (5 items)**

- Watching the Super Bowl was interesting.

- Watching the Super Bowl was pleasant.

- Watching the Super Bowl was fun.

- I felt good after watching the Super Bowl.

- I felt excited after watching the Super Bowl.

* 7-point Liker-type scales; anchors: 1 = strongly disagree, 7 = strongly agree.

**Extrinsic Rewards (3 items)**

After watching the Super Bowl…

- I felt more accepted by my peers.

- I felt more approved of by the people around me.

- I felt more included in social circles.

- I fit in better with others.

* 7-point Liker-type scales; anchors: 1 = strongly disagree, 7 = strongly agree.

**Consumer Satisfaction (4 items)**

- I am very satisfied with my experience watching the Super Bowl.

- My choice to watch the Super Bowl was a wise one.

- Really, I have enjoyed watching the Super Bowl.

- I don’t regret watching the Super Bowl at all.

* 7-point Liker-type scales; anchors: 1 = strongly disagree, 7 = strongly agree.

**Performance Satisfaction (1 item)**

- The performance of the team I supported was [very-dissatisfactory – very satisfactory]

* 7-point semantic differential scale.
